# Low Vitamin D Status Is Associated with Systemic and Gastrointestinal Inflammation in Dogs with a Chronic Enteropathy

**DOI:** 10.1371/journal.pone.0137377

**Published:** 2015-09-02

**Authors:** Helen F. Titmarsh, Adam G. Gow, Scott Kilpatrick, Jennifer A. Cartwright, Elspeth M. Milne, Adrian W. Philbey, Jacqueline Berry, Ian Handel, Richard J. Mellanby

**Affiliations:** 1 Royal (Dick) School of Veterinary Studies and The Roslin Institute, The University of Edinburgh, Roslin, Midlothian, EH25 9RG, United Kingdom; 2 Vitamin D Research Laboratory, Endocrinology and Diabetes, Institute of Human Development, University of Manchester, Manchester Academic Health Science Centre, Manchester Royal Infirmary, Manchester, M13 9WL, United Kingdom; University of Alabama at Birmingham, UNITED STATES

## Abstract

**Introduction:**

Vitamin D deficiency, as assessed by serum concentrations of 25 hydroxyvitamin D (25(OH)D), has been linked to the development of over-zealous and inappropriate inflammation in humans. However, the relationship between vitamin D status and inflammation in dogs is ill-defined. Chronic enteropathies (CE) are frequently diagnosed in client owned dogs, have a wide range of serum 25(OH)D concentrations, and represent a spontaneous model in which to probe the relationship between vitamin D and inflammation. The hypothesis of this study was that vitamin D status would be negatively associated with systemic and gastrointestinal inflammation in dogs with a CE. The aim of this study was to examine the relationship between serum 25(OH)D concentrations and markers of systemic and gastrointestinal inflammation in a cohort of dogs with CE.

**Methods and Materials:**

Serum 25(OH)D concentrations, together with neutrophil, monocyte, eosinophil and lymphocyte counts, duodenal histopathology scores, serum IL-2, IL-6, IL-8 and TNFα concentrations and were measured in 39 dogs with histologically confirmed CE. A linear regression model examined the relationship between serum 25(OH)D status and measures of inflammation.

**Results:**

Serum 25(OH)D concentrations were negatively associated with neutrophil and monocyte counts, duodenal histopathology scores and serum IL-2 and IL-8 concentrations. Dogs with low serum 25(OH)D concentrations typically had an inflammatory signature characterised by high monocyte and neutrophil numbers together with low lymphocyte numbers. There is a need to establish whether low vitamin D status is a cause or consequence of inflammation.

## Introduction

The traditional functions of vitamin D relate to its role in the maintenance of calcium homeostasis and bone metabolism. In recent decades, many cell types have been shown to express the vitamin D receptor, and the physiological roles of vitamin D have been shown to extend beyond skeletal metabolism [1, 2]. Consequently, numerous studies have examined the relationship between the immune response and vitamin D status as assessed by serum 25 hydroxyvitamin D (25(OH)D) concentrations in human patients. There is a growing body of evidence that vitamin D status is negatively associated with markers of inflammation, including circulating pro-inflammatory cytokines and acute phase proteins in a number of diseases including obesity [3, 4], inflammatory polyarthritis [5], diabetes mellitus [6], autoimmune diseases [7], inflammatory bowel disease [8, 9], and human immunodeficiency virus [10]. Furthermore, low vitamin D status has been associated with increased markers of inflammation in healthy humans [11–13].

The reasons why vitamin D status is negatively associated with inflammation is unclear. The vitamin D receptor (VDR) is found on most immune cells including macrophages, dendritic cells, T-lymphocytes and B-lymphocytes [14]. Vitamin D can promote immune tolerance by increasing T-regulatory cell populations [15], inhibiting the production of pro-inflammatory cytokines and increasing the production of anti-inflammatory cytokines [16–22].Vitamin D is also known to enhance the innate immune response to bacteria, by increasing the production of anti-microbial peptides such as cathelicidin [18, 23].

The relationship between vitamin D status and inflammation is poorly understood in dogs. Serum 25(OH)D concentrations are commonly reduced in a number of inflammatory diseases in dogs including congestive heart failure [24], *Spirocerca lupi* infections [25], protein losing enteropathy [26, 27] and renal disease [28]. Low vitamin D status has been negatively associated with C-reactive protein (CRP) in dogs with haemoabdomen [29]. However, other studies have reported a positive association between vitamin D and CRP concentrations in racing sled dogs [30]. Consequently, further work is needed to clarify the relationship between vitamin D and inflammation in dogs.

Dogs with chronic enteropathies (CE) represent a spontaneous model in which to explore the relationship between vitamin D status and inflammation. We have previously demonstrated the dogs with CE can have a range of vitamin D concentrations, ranging from profound deficiency to sufficiency [26, 31]. However, the relationship between 25(OH)D concentrations and inflammation in dogs with CE is poorly understood. A number of markers of systemic inflammation, including serum cytokines and leukocyte profiles can be measured in dogs [32]. In addition, the WSAVA gastrointestinal histopathology scoring system for endoscopic biopsies [33] provides a standardized means of assessing the extent of inflammation within gastrointestinal biopsies from dogs with a CE.

The hypothesis of this study was that vitamin D status would be negatively associated with local and systemic markers of inflammation in dogs with a CE. The objectives were to investigate the relationship between serum concentrations of 25(OH)D and blood neutrophil, lymphocyte, monocyte and eosinophil numbers, duodenal histopathology inflammation scores, and serum cytokine concentrations in dogs with histologically confirmed CE.

## Material and Methods

The records of dogs referred to the Hospital for Small Animals, Royal (Dick) School of Veterinary Studies for investigation of chronic gastrointestinal disease of more than three weeks in duration were retrospectively reviewed. Inclusion criteria including the following clinical signs, common reported in dogs with CE: vomiting, diarrhoea, increased borborygmi, abdominal pain, increased or decreased appetite and weight loss and also that the dogs had histopathological evidence of inflammation within the small or large intestine. Furthermore, there were no clinically significant abnormalities detected on hematology, biochemistry or abdominal ultrasonography indicative of non-gastrointestinal diseases for any of the dogs enrolled. In addition, the faeces of all dogs were negative for both helminth and *Giardia* infections.

Haematology variables, which included total white blood cell count, mature neutrophils, band neutrophils, lymphocytes, monocytes, eosinophils, basophils, total red blood cell counts, packed cell volume, haemoglobin, mean cell volume, mean cell haemoglobin concentration and platelet number were measured on ADVIA(r) 2120i System with Autoslide (Siemens Medical Solutions Diagnostics Ltd California, USA). At least a 100-white blood cell manual differential count was undertaken to establish the concentrations of neutrophils, monocytes, lymphocytes, eosinophils and basophils. Blood smears were evaluated under the direct supervision of a Board-certified veterinary clinical pathologist in every case. Biochemistry variables were measured on an ILab650 biochemistry analyser, (Diamond Diagnostics, USA). The area of intestinal tract which was biopsied (duodenum or duodenum and colon) was at the discretion of the primary clinician managing the case and based on presenting clinical signs. A diagnosis of CE was made if there was histological evidence of intestinal inflammation and no underlying aetiology was identified by on abdominal ultrasound, faecal samples or blood tests.

Serum samples retained for 25(OH)D measurement were frozen after being used for routine biochemical analysis. They were stored at –70°C before being sent to the laboratory for analysis on dry ice. Serum concentrations of 25(OH)D were measured as previously described in detail[34, 35]. Samples were extracted using acetonitrile and applied to C18 Silica Sep-paks. Separation of metabolites was by straight phase high performance liquid chromatography (HPLC) (Waters Associates, Milford, MA, USA) using a Hewlett-Packard Zorbax-Sil Column (Hichrom, Reading, UK) eluted with hexane:propan-2-ol:methanol (92:4:4). Serum 25(OH)D_2_ and 25(OH)D_3_ were measured separately by application to a second Zorbax-Sil Column eluted with hexane:propan-2-ol (98:2) and quantified by ultraviolet absorbance at 265 nm and corrected for recovery (sensitivity 5 nmol/L, intra- and inter-assay coefficients of variation 3·0% and 4·2%, respectively) [36]. This Supraregional Assay Service laboratory is accredited by CPA UK (CPA number 0865) and has been certified as proficient by the international Vitamin D Quality Assurance Scheme (DEQAS). Total 25(OH)D was defined as the sum of 25(OH)D_2_ and 25(OH)D_3._

Canine pro-inflammatory cytokines (IL-2, IL-6, IL-8 and TNF-α) were measured from serum samples using a multiplex electrochemiluminescence immunoassay system (Meso Scale Discovery; MSD) as previously described[32]. Assay diluent (25 μL) was added to all wells, plates sealed and incubated for 30 min at room temperature on an orbital shaker (600 rpm). Samples and standards, diluted in assay diluent, were added at 25 μL per well. Plates were again sealed and incubated for a further 2 hours at room temperature with shaking. At the end of the incubation period, wells were washed three times with 200 μL phosphate-buffered saline (PBS), supplemented with 0.05% Tween 20 (Sigma–Aldrich) for 30 s, then discarded. Detection antibody was added at 25 μL per well, plates sealed and incubated for a further 1 h at room temperature with shaking. Plates were washed three times and 150 μL of MSD Read Buffer added to each well, then electrochemiluminescence measured using the MSD Sector Imager 2400 plate reader.

A single veterinary pathologist (AP) reviewed the histopathological samples. The degree of inflammation present was assessed using a qualitative scoring system (WSAVA Standards for the Diagnosis of Gastrointestinal Inflammation in Endoscopic Biopsy Samples) [33]. Parameters which are assessed using this system include the following: histological changes (villous stunting, epithelial injury, crypt distension, lacteal dilatation, and mucosal fibrosis) and inflammatory infiltrates (intra-epithelial lymphocytes, lamina propria lymphocytes and plasma cells, lamina propria eosinophils, and lamina propria neutrophils). Changes were graded as normal (0), mild (1), moderate (2) or severe (3). The sums of all these parameters were added together to determine an intestine inflammatory score which ranged from 0 (normal) to 30 (very severe).

In order to investigate the relationship between multiple inflammatory markers and serum 25(OH)D concentrations, individual variables were examined using histograms and parameters which were not normally distributed (Anderson-Darling test) were transformed. Log10 transformation was undertaken except for square root transformation of eosinophil numbers since some eosinophil numbers were zero. The relationship between age, sex and 25(OH)D was examined using scatter plots and linear regression model. The relationship between 25(OH)D and inflammatory parameters was investigated using linear regression models. Age and sex was included in initial models in order to examine for any confounding effects. Final models were selected by removing potential confounders if their removal did not worsen model fit as assessed by the Akaike information criterion (AIC, a penalty parameter penalised measure of model fit). As the study wanted to examine whether 25(OH)D concentrations correlated to an inflammatory signature, medoid based partitioning was used to assign haematology parameters to three clusters. Three clusters was chosen on the basis of a consensus of results from 30 cluster count algorithms [37]. These clusters were identified independently of 25(OH)D concentrations. A Kruskal Wallis test was used to determine if there was difference in 25(OH)D concentrations between the three clusters [38]. Tukey and Kramer posthoc test for pairwise comparisons were then used to identify which clusters differ. Data was collected using Excel and analysed using R statistical software system [39]. A p value of <0.05 was used to define statistical significance.

The University of Edinburgh’s Veterinary Ethical Review Committee approved the study. Informed consent for the storage and subsequent use of residual clinical blood samples for research purposes was obtained at admission for each dog enrolled.

## Results

Thirty-nine dogs with a diagnosis of chronic enteropathies were enrolled. Breeds enrolled included; Boxer (9), cross breed (5), Labrador (2), Lurcher (2), Springer Spaniel (2), Cavalier King Charles Spaniel (2), Staffordshire Bull Terrier (2), and one of each of the following breeds; Irish Setter, Italian Greyhound, Toy Poodle, Shar-pei, Yorkshire Terrier, Pyrenees Mountain Dog, Hungarian Vizsla, Greyhound, German Short Haired Pointer, West Highland White Terrier, Cocker Spaniel, Dogue de Bordeaux, Shetland Sheep dog, Border Collie and a Border Terrier. The age range of dogs included was 6 to 136 months (median 65 months). Haematology, biochemistry, faecal parasitology and abdominal ultrasonography did not reveal any significant clinical abnormalities in any of the 39 dogs. Sixteen dogs underwent upper gastrointestinal endoscopy and 23 dogs had both upper and lower gastrointestinal endoscopy. Thirty- two dogs had duodenal biopsy samples which were available for histological review by a veterinary pathologist who was blinded to the clinical history and vitamin D status of the dogs. Only 32 samples were available for review as the remainder could not be retrieved from archived stores. Cytokine analysis was available in 23 cases as archived serum samples were not available for all cases.

**Fig 1:**
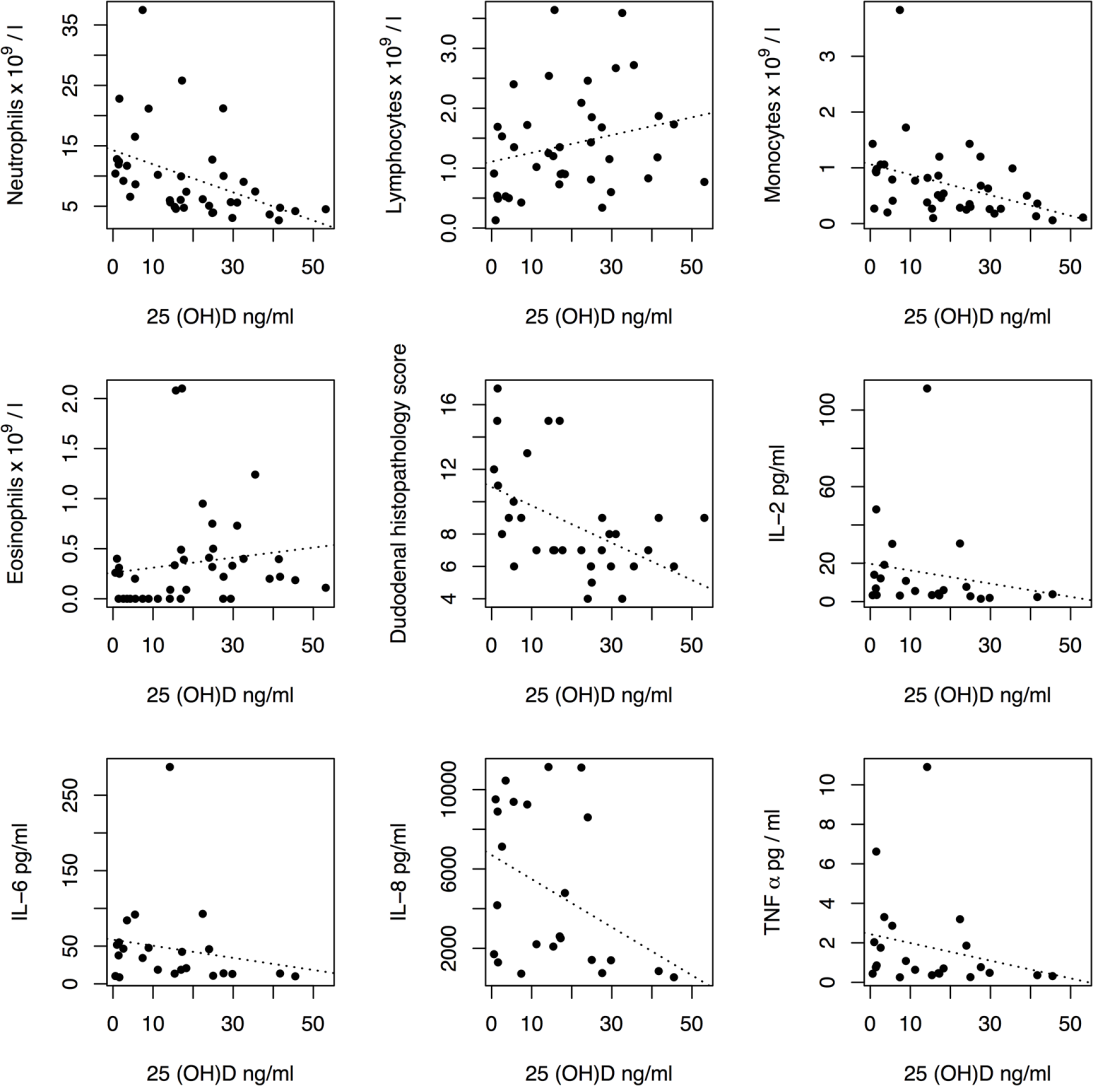
Relationship between haematology, serum cytokines and duodenal inflammatory score and serum 25(OH)D concentrations.

**Fig 2:**
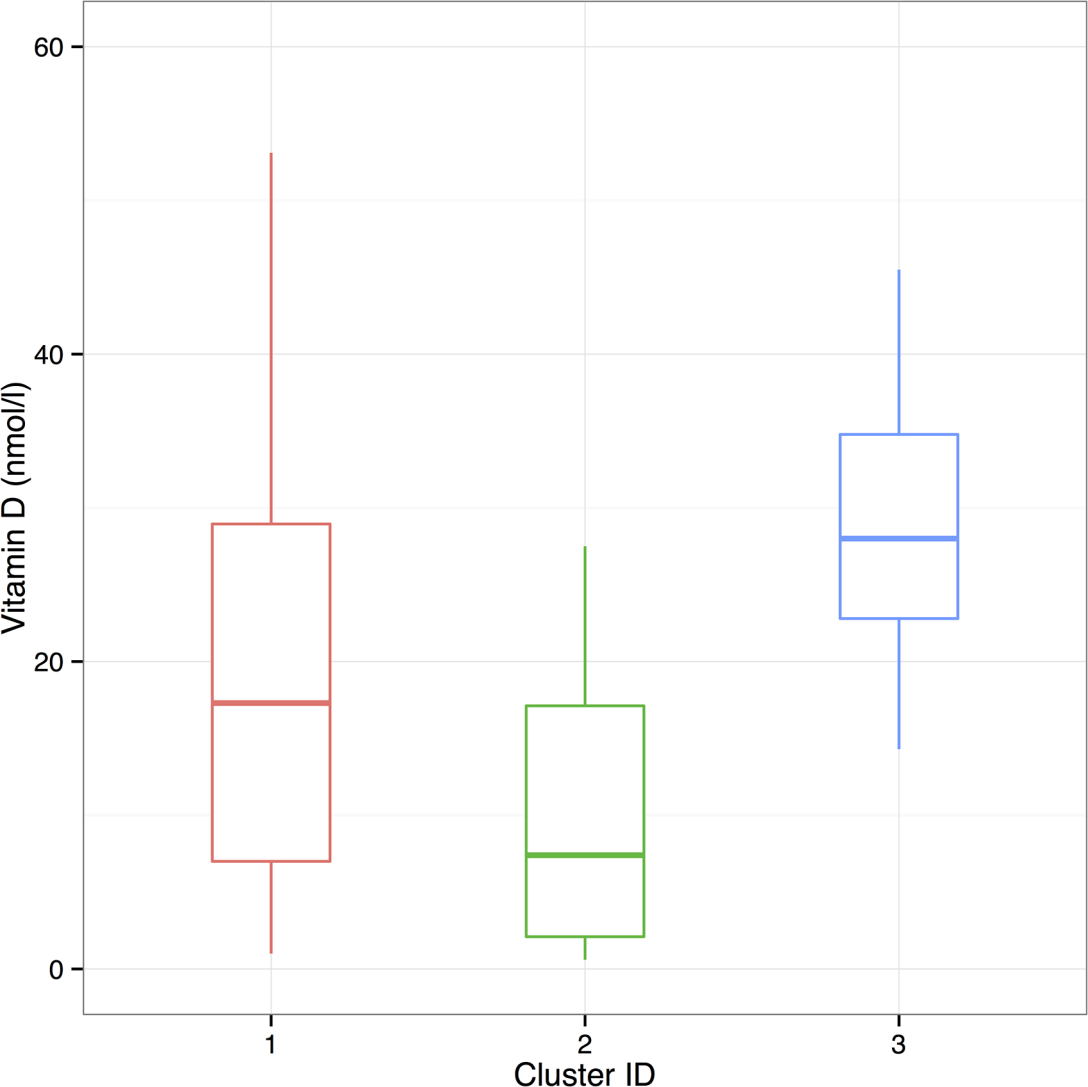
Box and whisker plot of serum 25(OH)D concentrations by cluster. The box extends from 25% to 75% percentile with the median and the whiskers extend to limits of the data.

**Fig 3:**
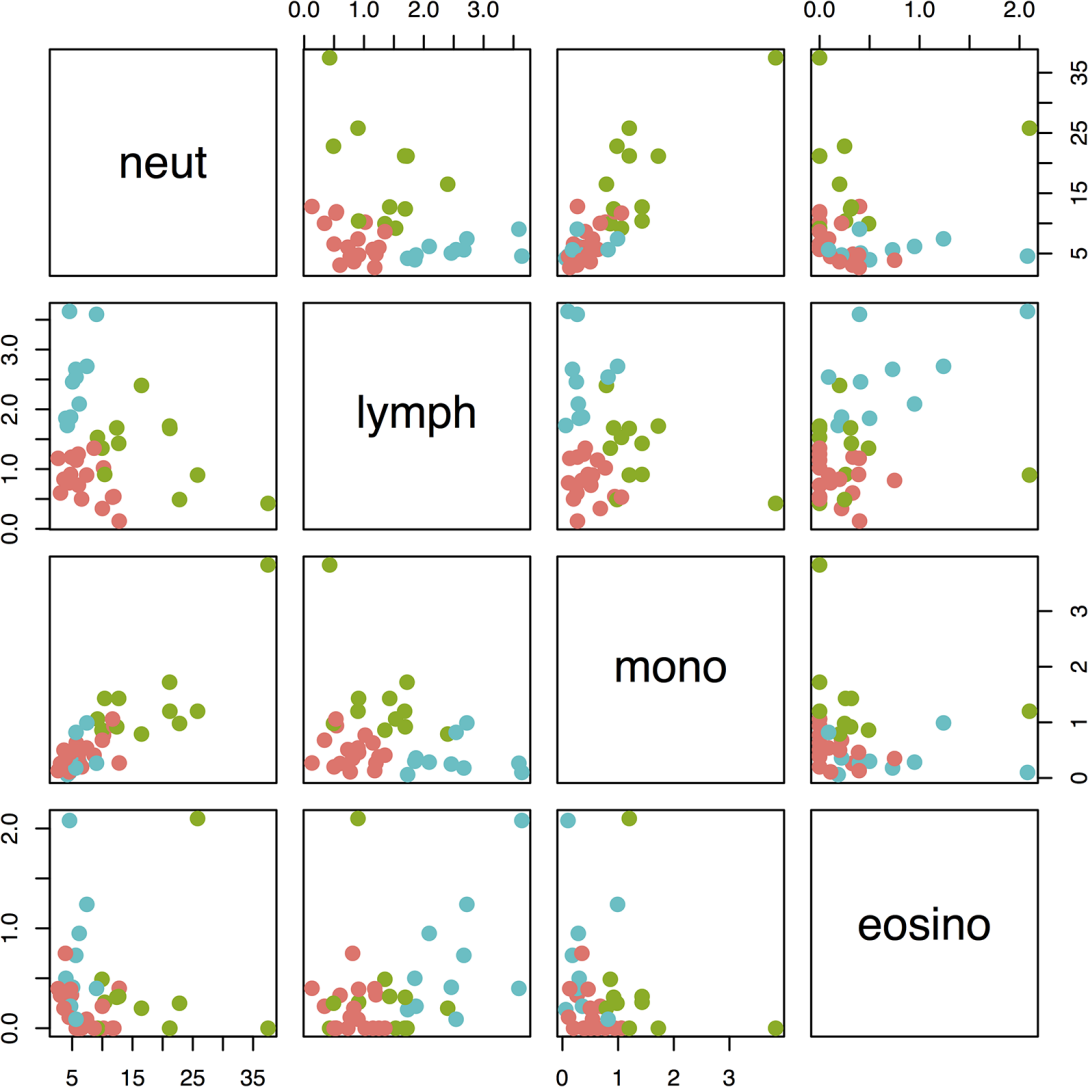
Pair-wise scatter plot of number of neutrophils (neut), monocytes (mono), eosinophils (eosino) and lymphocytes (lymph) labelled by cluster (red cluster 1, green cluster 2, blue cluster 3). Values on x and y axis denote cell concentrations x10^9^/l.

Age and sex were not predictive of serum 25(OH)D concentrations in a linear regression model. The relationships between serum 25(OH)D concentrations and haematology, duodenal inflammation score and serum cytokine results are shown in Fig 1. Neutrophil and monocyte counts, duodenal histopathology score and serum IL-2 and IL-8 concentrations were negatively associated with 25(OH)D concentrations (Table 1). Three clusters were identified in the haematology data (Figs 2 and 3). There was a significant difference in 25(OH)D concentrations between the three clusters (p = 0.009). Post-test analysis revealed a significant difference between clusters 2 and 3 (p = 0.006).

**Table 1:** Results of logistic regression model of inflammatory parameters on serum 25(OH)D concentrations.

Variable	Co-efficient (SE)	P value
Neutrophil number (log10)	-0.011 (0.003)	<0.001 *
Monocyte number (log10)	-0.016 (0.004)	<0.001 *
Eosinophil number (sqrt)	0.008 (0.004)	0.09
Lymphocyte number (log10)	0.005 (0.004)	0.16
Duodenal histopathology score (log10)	-0.012 (0.004)	0.006 *
IL-2 (log10)	-0.015 (0.007)	0.048 *
IL-6 (log10)	-0.011 (0.006)	0.09
IL-8 (log10)	-0.018 (0.006)	0.01 *
TNF-α (log10)	-0.014 (0.007)	0.06

*denotes statistical significance P<0.05

## Discussion

The central finding of this study is that serum 25(OH)D concentrations negatively correlate with systemic markers of inflammation including neutrophil and monocyte counts, serum IL-2 and IL-8 concentrations and local inflammation as measured by duodenal histopathology scores. Linear regression and medoid based partitioning analysis demonstrated that dogs with low vitamin D status had an inflammatory signature consisting of high neutrophil and monocyte counts and low lymphocyte numbers. A typical stress and/or inflammatory leukogram in dogs consist of neutrophilia, monocytosis and lymphopenia. These changes provide evidence of an association between reduced serum 25(OH)D concentrations and systemic inflammation in this population of dogs.

The results of this study also show that IL-2 and IL-8 concentrations are increased in the serum of dogs with CE and associated with lower 25(OH)D concentrations. Interleukin-2 acts on activated lymphocytes resulting in their expansion, and differentiation into cytotoxic lymphocytes, natural killer cells and T helper cells [40]. Interleukin-2 is under direct transcriptional regulation by 1,25(OH)_2_D and can decrease IL-2 production [41]. Culture of T lymphocytes with vitamin D from patients with systemic lupus erythematous decreased production of IL-2 [42]. Similarly, vitamin D supplementation has been associated with decrease in circulating IL-2 concentrations in children with atopy [43]. In addition, increased IL-2 concentrations have been reported in vitamin D insufficient adults [44]. Serum IL-8 concentrations were also increased in this population of dogs with CE and lower 25(OH)D concentrations. In human IBD, IL-8 is an important inflammatory mediator, playing a role in the initiation and maintenance of IBD by recruiting neutrophils into the inflamed gastrointestinal tract [45]. An increase in serum IL-8 concentrations have also been reported in human patients with IBD [46]. Vitamin D has been shown to exert anti-inflammatory effects by decreasing IL-8 production in intestinal cell cultures [47]. Our results also indicate that low serum vitamin D concentrations are associated with more severe inflammation as assessed by duodenal histopathology scores. This provides evidence of an association between intestinal inflammation and reduced vitamin D concentrations.

It is unclear if the low 25(OH)D concentrations observed in inflammatory diseases are causally related to inflammation or if lower vitamin D status occur as a consequence of the inflammatory process itself. Serum 25(OH)D concentrations have been shown to decrease following elective surgical procedures in human patients, coupled with increases in inflammatory markers such as CRP, suggesting vitamin D is a negative acute phase reactant [48]. Serum 25(OH)D concentrations also fall rapidly in other inflammatory conditions such as acute pancreatitis [49]. Increases in pro-inflammatory cytokines have been proposed to be the cause of decreases in serum 25(OH)D in acute inflammation. For example, changes in serum 25(OH)D over several weeks corresponded with increases in serum pro-inflammatory cytokines, including TNF-α, IFN-γ, IL-1β, GM-CSF and IL-6 concentrations, in patients undergoing knee arthroplasty [50]. However, not all studies looking at illness events associated with acute inflammation have demonstrated decreases in serum 25(OH)D concentrations [51, 52]. In addition, changes in vitamin D status following inflammation may relate to changes in vitamin D binding proteins. For example, serum vitamin D binding protein decreases in the face of acute inflammation [53].

Numerous studies have investigated the potential benefit of vitamin D supplementation in a range of inflammatory diseases. In ill patients, vitamin D administration reduces some markers of inflammation in early chronic kidney disease [54], end-stage renal disease [55], congestive heart failure [56], systemic lupus [57] and colorectal adenoma [58]. In addition markers of inflammation decreased in elderly women with vitamin D insufficiency in response to supplementation with a mega-dose of vitamin D3 [59]. However, other studies investigating the effects of vitamin D supplementation on markers of inflammation have failed to demonstrate *in–vivo* anti-inflammatory effects. For example, high dose vitamin D treatment did not result in a decrease in inflammatory markers in people with low vitamin D status diagnosed with: pre-diabetes and type 1 diabetes [6, 60], hypertension [61] and urticaria [62]. Furthermore, increases in pro-inflammatory cytokines were documented in patients with osteoporosis after vitamin D supplements were administered [63]. There are many potential explanations for why the results of different studies reported differing effects of vitamin D supplementation on markers of inflammation. These include differences in the duration for which vitamin D supplementation was given, the type of vitamin D used for supplementation and the effects of various diseases. In one meta-analysis it appears that the benefits of vitamin D in reducing inflammatory markers is dependent on the disease state studied and initial, pre-treatment 25(OH)D concentrations [64]. The benefits of vitamin D supplementation appear to be most evident in markedly inflammatory diseases where serum 25(OH)D concentrations are initially low.

Although hypovitaminosis D in CE has traditionally been considered to be the result of intestinal disease, there is growing evidence that hypovitaminosis D may contribute to the initiation of intestinal inflammation. Intestinal epithelial vitamin D receptor is important in the regulation of mucosal inflammation by maintaining the integrity of the mucosal barrier [65], suggesting hypovitaminosis D may also perpetuate inflammation. However, the expression of VDR is influenced by mucosal inflammation and is down-regulated by mucosal pro-inflammatory cytokines [65, 66]. Studies in experimental models of inflammatory bowel disease have demonstrated that vitamin D can have an anti-inflammatory effect. For example, in a murine model of IBD, 1,25(OH)_2_D was shown to inhibit a number of genes involved in regulating TNF-α production and signaling [67]. Furthermore, VDR knockout mice produce significantly higher cytokine concentrations in response to chemically induced gastrointestinal inflammation than wild type mice [68]. These findings suggest that vitamin D is important in regulating gastrointestinal inflammation. However, it is not clear whether these findings can be extrapolated directly to patients with IBD. For example, CRP, ESR, TNF-α, IL-17, IL-10 and vascular endothelial growth factor concentrations did not change following vitamin D_3_ supplementation in people with IBD [69]. Therefore, further work is needed to determine the role of vitamin D in the treatment of inflammatory bowel disease in people and dogs.

There are some limitations to this study. The retrospective nature of this study made it impossible to standardise the drugs and dietary treatments the dogs received prior to referral. In addition, serum cytokine concentrations and gastrointestinal histology slides were not available from all dogs.

## Conclusion

In summary, this study has shown that serum 25(OH)D concentrations are inversely associated with markers of systemic inflammation in dogs with CE, and with severity of inflammatory changes seen in histopathology samples. Further studies are required to assess whether low vitamin D status is a cause or a consequence of inflammation.
